# Investigation of TNBC *in vitro* Antiproliferative Effects of Versatile Pirrolo[1,2-a]quinoxaline Compounds

**DOI:** 10.3389/fmolb.2019.00012

**Published:** 2019-03-12

**Authors:** Mariarita Perri, Francesca Aiello, Erika Cione, Gabriele Carullo, Luisa Amendola, Sarah Mazzotta, Maria Cristina Caroleo

**Affiliations:** ^1^Department of Pharmacy Health and Nutritional Sciences, University of Calabria, Rende, Italy; ^2^Química Orgánica Y Farmacéutica, Universidad de Sevilla, Seville, Spain

**Keywords:** pyrroloquinoxalines, triple-negative breast cancer, autophagy, antiproliferative activity, one-pot reaction

## Abstract

The triple-negative breast cancer (TNBC) is characterized by a more aggressive nature and poorer prognosis, nowadays none pharmaceutical approach is still available. For this reason, the research of new active compounds and attractive targets represents an interesting field. In this context MDA- MB-231 cell line was selected to evaluate the antiproliferative effects of new [1,2-a]-pyrroloquinoxaline derivatives. The MTT assay revealed that the amine forms of synthesized molecules were more active compared to iminic ones at 72 h of incubation. The antiproliferative effect of the most promising compounds highlighted the formation of autophagic vacuoles.

## Introduction

Breast cancer (BC) is a heterogeneous and complex disease and one of the leading causes of death in 40–59 years old women. An estimated 1 million cases of BC are diagnosed annually worldwide and of these, about 170,000 possess a triple negative (TNBC) phenotype (Plasilova et al., 2016). TNBC is characterized by low estrogen and progesterone receptors expressions as well as low HER2/Neu amplification. TNBC is shows a high rate of early recurrence and distant metastasis (Al-Mahmood et al., 2018). Given the aggressive nature of TNBC an accurate diagnosis is of pivotal importance for optimal therapy and prognosis. While ER+ and PR+ and HER2/Neu+ BC are responsive to hormone or targeted therapy respectively, TNBC is still an orphan disease in terms of therapeutic progress. Treatment options beyond the conventional chemotherapy include EGFR, VEGF, and PARP inhibitors (Dawson et al., 2009). These last have shown a strong component of nitrogenous nuclei. Studies performed on TNBC cell line have shown an antiproliferative activity for molecules endowing a pyrroloquinoxaline nucleus, the pyrrolo[2,3-b]quinoxalines. In addition, SAR studies, based on new pyrrolo[1,2-a]quinoxalines, demonstrated the importance of substitution in the C-4 position of the pyrrolequinoxaline nucleus and the need for a functionalization of the pyrrole ring (Prasad et al., 2013). Therefore, other studies were performed on breast cancer cell lines expressing the G protein-coupled estrogen receptor (GPER o GPR30) and was evaluated and attributed an antiproliferative activity to molecules with pyrrole[1,2-a]quinoxaline structure (Aiello et al., 2017).

In the present study (Amendola, 2018), the antiproliferative activity of new molecules with pyrrole[1,2-a]quinoxaline scaffold, on TNBC cell line MDA-MB-231 was evaluated. The aminic/iminic form of all the new compounds were obtained by a One-Pot reaction. Results from MTT assay, revealed that the amine forms are more active compared to iminic ones. A significant decrease of cell viability was achieved at 72 h, highlighting the formation of autophagic vacuoles stained by monodansylcadaverine (MDC).

## Materials and Methods

### General Synthetic Procedure

All reagents used in this synthesis, equipment, and spectroscopic data are reported in the Supplementary Information. In a round-bottom flask containing 5 mL ethanol (96°), *p*-toluensulphonic acid (0.13 mmol) and the appropriate aldehyde (1.26 mmol) were mixed. After 5 min, 2-(1H-pyrrol-1-yl) aniline (1.26 mmol) was added. The resulting mixture was stirred for 30 min at room temperature. Then, the solvent was removed under reduced pressure. The residue was dissolved in ethyl acetate (50 mL) and extracted with a 5% aqueous solution of NaHCO_3_ (3 x 50 mL). The combined organic layers were made anhydrous with Mg_2_SO_4_, filtered and concentrated under reduced pressure. The pure compounds were obtained after purification with column chromatography using a mixture *n*-hexane: ethyl acetate as eluent.

### Cell Culture and MTT Proliferation Assay

The human epithelial breast carcinoma MDA-MB-231 cells were purchased from the American Type Culture Collection (ATCC). Cells were cultured in Dulbecco's modified Eagle's medium (DMEM) (Corning, Cellgro) supplemented with 10% fetal bovine serum (FBS) (Invitrogen) and maintained in a humidified 5% CO_2_ incubator at 37°C as recommended by ATCC. Cell viability was determined by measuring the reduction of 3-(4, 5-dimethylthiasol-2-yl)-2, 4,- diphenyltetrazolium bromide (MTT) by mitochondrial succinate dehydrogenase. Briefly, cells were seeded at 1 × 10^4^ and incubated with various concentrations of compounds **L1-10** (1–20 μM) alone or with DMSO (as a vehicle) in 96-well plates for 24 and 72 h. The optical density (OD) was calculated as the difference between the absorbance at the reference wavelength (620 nm) and the absorbance at the test wavelength (570 nm). Percent viability was calculated as (OD of drug treated sample/OD of control) ×100.

### DAPI and MDC Staining

Changing in morphology for nuclei swelling or autophagic vacuoles formation were assessed by 4,6-diamidino-2-phenylindole (DAPI) or monodansylcadaverine (MDC) staining. 1 x 10^5^ MDA-MB-231 cells were grown on covers lip in 24-well plates and treated with **L1, L5**, and **L6** at 20 μM at 72 h. Cells were fixed with 4% paraformaldehyde (ThermoScientific) and imaged using by a fluorescent microscope. At least tree visual fields were analyzed under fluorescence microscope (Leica FL560 fluorescence microscopy) for each sample.

### Statistical Analysis

Data were expressed as mean ± standard deviation from three independent experiments ran in triplicate. Statistical differences were determined by one-way analysis of variance (ANOVA) followed by Tukey's Multiple Comparison Test. Differences were considered statistically significant for *p* < 0.05 (^*^), *p* < 0.01 (^**^), *p* < 0.001 (^***^).

## Results

### One-Pot Synthesis of Pyrrolequinoxaline Derivatives

**Scheme 1:**
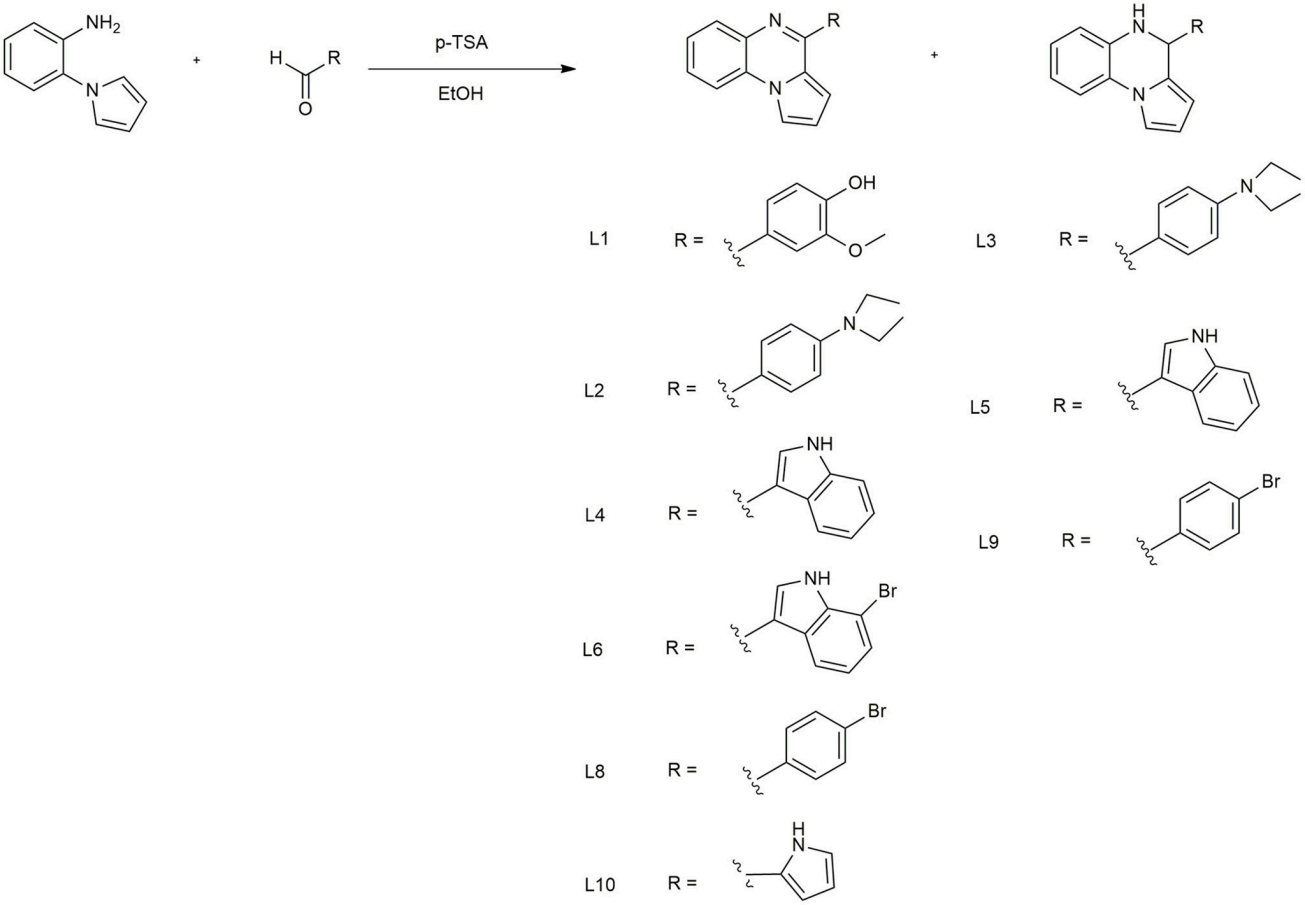
Synthetic method to obtain the pirrole[1,2-a]quinoxalines **L1-10**.

The synthesis of pirrolo[1,2-a]quinoxalines **L1-10** (Scheme 1) was carried out according to a very efficient one-pot reaction. (Preetam and Nath, 2015; Aiello et al., 2017) that allows to obtain both aminic/iminic form for some of all prepared compound. Particularly, for **L6, 8, 9** it was registered the formation of the iminic form only. The characteristic signals of the diverse structures, used to verify which form were obtained were the -NH (5.20–5.30 ppm) and -CH (5.10–5.20 ppm) of the aminic form. Complete spectroscopic data are reported in Supplementary Information.

### Antiproliferative Activity of Pyrrolo[1,2-a]Quinoxaline Derivatives on TNBC

**Figure 1:**
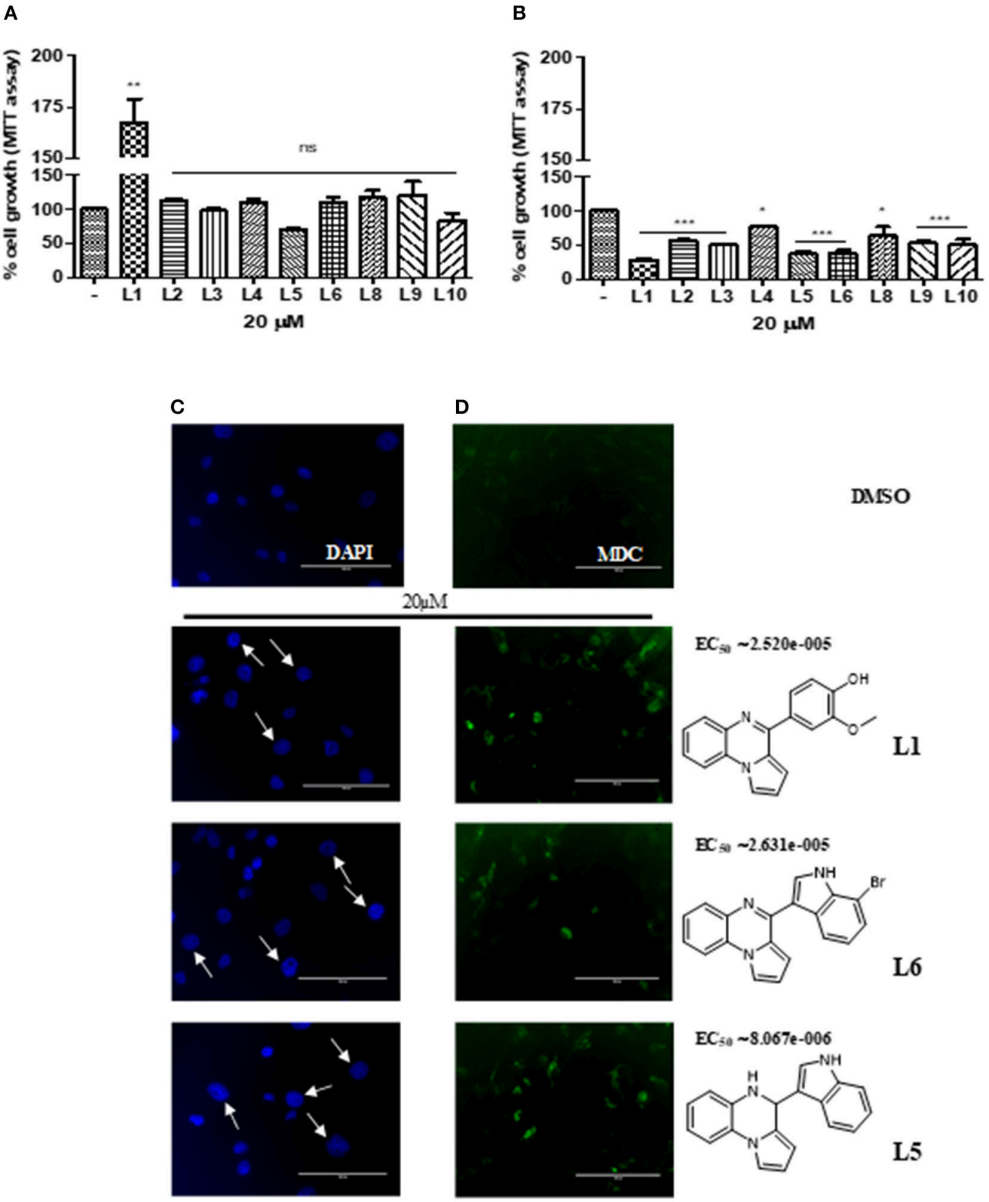
**(A)** Cell viability of L1-L10 compounds at 20 μM on MDA-MB231 at 24 h of incubation and **(B)** at 72 h. **(C)** Nuclear swelling indicated by white arrows and stained with DAPI. **(D)** Autophagic activity labeling vacuoles which exhibit lysosomal activity by MDC.

To determine whether the new derivatives provide the desired TNBC antiproliferative activity, MDA-MB-231 cell line, were exposed to several concentration of **L1-6 L8-10** for 24 or 72 h and then cell viability was assessed by MTT assay Figures 1A,B. Although the small number of compounds, the *in vitro* results indicate the impact of the different substituents on the anti-proliferative activity. As shown in Figure 1A, the compound **L5**, that is the aminic form of **L6** with an indole substituent on C4 position, inhibited the cell proliferation at 24 h, whereas the other compounds were ineffective, out contrary **L1**, bearing a vanillic residue on C4, induced proliferation. On the other hand, at 72 h all the synthetic compounds highlighted a decrease of the proliferation rate, including **L1** (Figure 1B). Particularly, **L1, 5 and 6**, resulted in a potent cytotoxicity effect which was able to induced nuclear swelling stained with DAPI Figure 1C suggesting autophagic cell death. To confirm this hypothesis, autophagic cell activity was evaluated by labeling vacuoles with MDC dye. We appreciated, positive labeling by MCD as shown in Figure 1D. EC_50_ was calculated with GraphPad Prism 5.0 using the non-linear regression curve fit. To straight our observations **L1,5,6** were tested on MDA-MB-468 cell line, pointing out a vitality decreasing of 36, 40, and 41% respectively.

## Discussion

Autophagy is a self-eating behavior initiated by cells as a protective and pro-survival pathway against DNA damage as well as by metabolic and therapeutic stress. When excessive this process can lead to cell death in many type of cancers including breast (Perri et al., 2010, 2018). To the best of our knowledge, the *in vitro* results obtained in this study, it is possible to confirm the versatility of the pyrroloquinoxaline nucleus that once again showed interesting antiproliferative activity assessed with MTT assay. The decrease in vitality is due to the induction of autophagy in TNBC as it is evident by DAPI and MDC staining. In fact, this latter staining highlighted cells autophagic vacuoles formation after treatment with **L1, 5**, and **6** at 72 h. These three compounds show important chemical differences. Firstly, **L1** presents a vanillic residue on C4 position, conversely to **L5, 6**, an aminic and iminic form respectively, that bearing both an indole nucleus, and in the case of **L6** also with a bromine atom in position C7 of indole moiety. Vanillic and indole are both privileged natural scaffolds, able to confer important antiproliferative properties, that can work synergistically when are linked at a pyrroloquinoxaline nucleus, already consolidated building blocks for active and promising anticancer agents (Aiello et al., 2017). Further, the synthesis of new molecules is in progress aimed to verify if the indole, on C4 position of pyrrolo[1,2-a]quinoxaline scaffold, differently decorated with other substituents, improves the final antiproliferative effect.

## Data Availability

The raw data supporting the conclusions of this manuscript will be made available by the authors, without undue reservation, to any qualified researcher.

## Author Contributions

FA and EC: conception and study design, manuscript preparation; MP: biological assays and results recording; LA, GC, and SM: bibliographic research, synthesis, and chemical characterization of new compounds; MC: critical reading and intellectual assessment of manuscript. All authors read and approved the final manuscript.

### Conflict of Interest Statement

The authors declare that the research was conducted in the absence of any commercial or financial relationships that could be construed as a potential conflict of interest.

## Supplementary Material

Data Sheet 1 Supplementary experimental description and NMR characterization of pyrroloquinoxaline derivatives.
