# Supplementary material for: Investigation of TNBC in vitro Antiproliferative Effects of Versatile Pirrolo[1,2-a]quinoxaline Compounds
Source: Front Mol Biosci. 2019 Mar 12;6:12. doi: 10.3389/fmolb.2019.00012 (PMC6423061; doi:10.3389/fmolb.2019.00012)
Supplement: Data Sheet 1 — Supplementary experimental description and NMR characterization of pyrroloquinoxaline derivatives. [file Data_Sheet_1.pdf]

Supplementary Material

1 Supplementary Data

General

All reagents were purchased from Sigma-Aldrich and used without further purification. ^{1}H NMR and ^{13}C NMR spectra were recorded on a Bruker Advance 300 (^{1}H: 300 MHz, ^{13}C: 75 MHz). The chemical shifts (*δ*) are expressed in parts per million (ppm) while the coupling constants (*J*) in hertz (Hz). Flash column chromatography purifications were performed using glass columns with Merck silica gel (0.040–0.063 mm). TLC analyses were performed on Merck silica gel 60 F254 plates and visualized by UV light at 254 and 366 nm of wavelength. Organic solutions were dried over anhydrous $NaSO_4$ and evaporated on a rotary evaporator Büchi R-II under reduced pressure. Melting points were obtained by a GallenKamp 21374 apparatus and were uncorrected. Mass spectra data were determined after electron impact ionization at 70 eV with HP 5973MS spectrometer. Yields refer to purified products and are not optimized.

2-methoxy-4-(pyrrolo[1,2-a]quinoxalin-4-yl)phenol (L1)

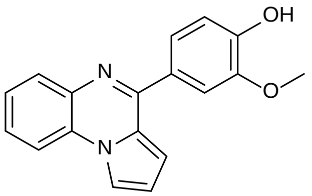

Obtained from 4-hydroxy-3-methoxybenzaldehyde as light brown resin. Yield 27 %. **^{1}H-NMR (300 MHz $CDCl_3$) :** δ (ppm) 8.04 (dd, 1H, *J* =1.5 Hz, 7.7 Hz) 8.02-8.00 (m, 1 H) 7.89 (dd, 1H, *J* =1.6 Hz, 8.2 Hz) 7.6 (d, 1H, *J* =1.9 Hz) 7.53 (dd, 1H, *J* =1.6 Hz, 7.3 Hz) 7.49 (t, 1H, *J* =2.1 Hz) 7.09-7.07 (m, 1H) 7.06-7.03 (m, 2H) 6.93-6.89 (m, 1H) 4.0 (s, 3H). **^{13}C-NMR (75 MHz $CDCl_3$) :** δ (ppm) 178.5, 147.5, 146.8, 136.2, 129.9, 127.2, 127.0, 125.3, 125.2, 122.3, 114.6, 114.2, 113.9, 113.5, 111.2, 108.7, 102.2, 56.1. ***m/z* :** 290 [M^+].

N,N-diethyl-4-(pyrrolo[1,2-a]quinoxalin-4-yl)aniline (L2)

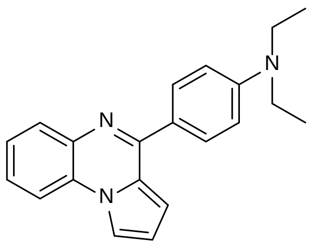

Obtained from 4-(diethylamino)benzaldehyde as dark red oil. Yield 30 %. **^{1}H-NMR (300 MHz $CDCl_3$) :** δ (ppm) 8.20-8.00 (m, 3H) 7.90-7.80 (m, 1H) 7.55-7.40 (m, 2H) 7.12-6.90 (m, 1H) 6.80-6.55 (m, 3H) 3.60-3.40 (m, 4H) 1.40-1.15 (m, 6H). **^{13}C-NMR (75 MHz $CDCl_3$) :** δ (ppm) 149.9, 129.8, 129.6 (x2C), 129.3, 128.4 (x2C), 127.7 (x2C), 126.6, 122.3, 119.2, 114.8, 114.4, 108.6, 60.4, 47.8 (x2C), 14.1 (x2C). ***m/z* :** 315 [M^+].

4-(4,5-dihydropyrrolo[1,2-a]quinoxalin-4-yl)-N,N-diethylaniline (L3)

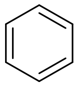

Obtained from 4-(diethylamino)benzaldehyde as red oil. **^{1}H-NMR (300 MHz $CDCl_3$) :** δ (ppm) 7.40-7.35 (m, 5H) 7.22-7.20 (m, 1H) 6.95 (t, 1H, *J* =2.5 Hz) 6.89-6.80 (m, 1H) 6.75-6.60 (m, 2H) 6.30 (t, 1H, *J* =3.2 Hz) 5.70 (bs, 1H, -NH) 5.40 (s, 1H, -CH) 3.60-3.35 (m, 4H) 1.49-1.28 (m, 6H). **^{13}C-NMR (75 MHz $CDCl_3$) :** δ (ppm) 149.9, 136.6, 136.5, 129.8, 129.5, 128.9, 125.8, 125.2, 125.1, 124.9, 122.3, 119.2, 114.8, 114.4, 113.6 (x2C), 60.4, 47.8 (x2C), 14.1 (x2C). ***m/z* :** 317 [M^+].

4-(1H-indol-3-yl)pyrrolo[1,2-a]quinoxaline (L4)

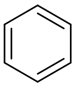

Obtained from 1H-indole-3-carbaldehyde. Yield 66 %. Spectroscopic data are in agreement with those reported in literature.

4-(1H-indol-3-yl)-4,5-dihydropyrrolo[1,2-a]quinoxaline (L5)

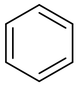

Obtained from 1H-indole-3-carbaldehyde as orange amorphous solid. Yield 82 %. **^{1}H-NMR (300 MHz $CDCl_3$) :** δ (ppm) 8.20 (bs, 1H) 7.60 (d, 1H, *J* =7.9 Hz) 7.43-7.33 (m, 2H) 7.29-7.10 (m, 5H) 6.99 (t, 1H, *J* =9.0 Hz) 6.9 (t, 1H, *J* =6.3 Hz) 6.72 (d, 1H, *J* =3.99 Hz) 6.28 (t, 1H, *J* =2.3 Hz) 6.90 (bs, 1H) 5.80 (s, 1H, –CH). **^{13}C-NMR (75 MHz $CDCl_3$) :** δ (ppm) 136.5, 136.2, 132.0, 129.8, 129.2, 126.6, 126.0, 125.8, 123.0, 121.6, 119.8, 119.7, 119.0, 114.2, 114.1, 111.3, 110.8, 67.0. ***m/z* :** 286 [M^+].

4-(7-bromo-1H-indol-3-yl)pyrrolo[1,2-a]quinoxaline (L6)

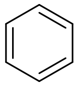

Obtained from 7-bromo-1H-indole-3-carbaldehyde as orange amorphous solid. Yield 18 %. **^{1}H-NMR (300 MHz $CDCl_3$) :** δ (ppm) 8.80 (bs, 1H) 8.45 (d, 1H, *J* =7.9 Hz) 8.05 (t, 3H, *J* =4.9 Hz) 7.9 (d, 1H, *J* =5.3 Hz) 7.50-7.35 (m, 3H) 7.20 (t, 1H, *J* =4.1 Hz) 7.10 (d, 1H, *J* =2.2 Hz) 6.92 (t, 1H, *J* =3.8 Hz). **^{13}C-NMR (74 MHz $CDCl_3$) :** δ

(ppm) 149.2, 135.1, 129.7, 127.6, 126.8, 126.2, 125.8, 125.4, 125.3, 125.1, 122.3, 121.6, 120.6, 114.4, 113.6, 113.5, 112.6, 107.5, 104.5. ***m/z* :** 361 [M^+].

4-(4-bromophenyl)pyrrolo[1,2-a]quinoxaline (L8)

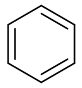

Obtained from 4-bromobenzaldehyde as gold oil. Yield 15 %. **^{1}H-NMR (300 MHz $CDCl_3$) :** δ (ppm) 8.10 (s, 1H) 7.90 (d, 1H, *J* =4.8 Hz) 7.69 (d, 1H, *J* =6.7 Hz) 7.62-7.6 (m, 1H) 7.40-7.30 (d, 1H, *J* =4.7 Hz) 7.10-6.6 (m, 4H) 6.30-6.20 (m, 1H). **^{13}C-NMR (75 MHz $CDCl_3$) :** δ (ppm) 140.5, 135.7, 131.7, 130.2, 129.5, 129.2, 128.4, 125.3, 124.7, 122.1, 119.5, 115.3, 114.7, 114.4, 110.2, 105.9. m/z : 323 [M^+].

4-(4-bromophenyl)-4,5-dihydropyrrolo[1,2-a]quinoxaline (L9)

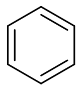

Obtained from 4-bromobenzaldehyde as gold solid. Yield 60 %. **^{1}H-NMR (300 MHz $CDCl_3$) :** δ (ppm) 7.90-7.89 (m, 1H) 7.60 (d, 1H, *J* =7.3 Hz) 7.55 (d, 2H, *J* =7.5 Hz) 7.30 (d, 2H, *J* =7.8 Hz) 7.29-7.20 (m, 1H) 7.02 (t, 1H, *J* =9.9 Hz) 6.89 (t, 1H, *J* =7.6 Hz) 6.77 (d, 1H, *J* =7.7) 6.30-6.26 (m, 1H) 5.60 (bs, 1H) 5.50 (s, 1H, -CH). **^{13}C-NMR (75 MHz $CDCl_3$) :** δ (ppm) 140.5, 135.8, 131.8, 130.2, 129.6, 129.3, 128.4, 125.4, 124.7, 122.1, 119.6, 115.4, 114.7, 114.5, 110.2, 106.0, 55.6. ***m/z* :** 326 [M^+].

4-(1H-pyrrol-2-yl)pyrrolo[1,2-a]quinoxaline (L10)

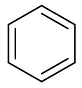

Obtained from 1H-pyrrole-3-carbaldehyde. Yield 34 %. Spectroscopic data are in agreement with those reported in literature.
